# Supplementary material for: Carbon sequestration during core formation implied by complex carbon polymerization
Source: Nat Commun. 2019 Feb 15;10:789. doi: 10.1038/s41467-019-08742-9 (PMC6377623; doi:10.1038/s41467-019-08742-9)
Supplement: Supplementary file 1 — Supplementary Information [file 41467_2019_8742_MOESM1_ESM.pdf]

Supplementary Information

# Carbon sequestration during core formation implied by complex carbon polymerization

Natalia V. Solomatova[*1], Razvan Caracas[1] and Craig E. Manning[2]

[1]Ecole Normale Supérieure de Lyon, Université Claude Bernard Lyon 1, Laboratoire de Géologie de Lyon, CNRS UMR 5276, Lyon, France.
[2]Department of Earth, Planetary and Space Sciences, University of California, Los Angeles, USA.

## Supplementary Note 1

A fourth-order Birch-Murnaghan equation of state[1], expressed as

$$P = \frac{3}{2}K_0\left(\left(\frac{V_0}{V}\right)^{\frac{7}{3}} - \left(\frac{V_0}{V}\right)^{\frac{5}{3}}\right)\left(1 + \frac{3}{4}(K' - 4)\left(\left(\frac{V_0}{V}\right)^{\frac{2}{3}} - 1\right)\right.$$

$$\left. + \frac{3}{8}\left(K_0K'' + (K' - 3)(K' - 4) + \frac{35}{9}\right)\left(\left(\frac{V_0}{V}\right)^{\frac{2}{3}} - 1\right)^2\right)$$

was used to fit our pressure-density data using the orthogonal distance regression method[2]. The effect of temperature and oxidation state is shown in Supplementary Fig. 1. We compare the zero-pressure density, zero-pressure isothermal bulk modulus ($K_0$) and bulk modulus derivative ($K'_0$) at high temperatures in Supplementary Table 1. $K_0$ is positively correlated with the degree of oxidation and negatively correlated with temperature and $CO_x$ concentration while $K'_0$ is positively correlated with the degree of oxidation, temperature and $CO_x$ concentration. The compressibility of the melt likely increases with increasing $CO_x$ due to the addition of oxygen, which is highly compressible relative to cations. The density difference between carbon-bearing pyrolite melt and carbon-free pyrolite melt decreases with increasing pressure, reaching a local minimum at ~30 GPa after which the density difference between the melts increases again. The larger density difference at pressures below 30 GPa compared to at pressures above 30 GPa suggests that carbon-bearing melts will be less buoyant at lower-mantle pressures than at pressures of the upper mantle.

## Supplementary Note 2

The formation of diamonds in Earth's deep mantle is poorly understood due to the limitations of experimental methods and our inability to access the diamond-forming depths of the mantle. Diamonds likely form in a wide range of pressure-temperature regimes and chemical environments[3,4,5]. In the uppermost mantle, diamonds are thought to form during metamorphic reactions[6], metasomatism[7,8], and in magmatic processes[3]. It has been proposed that many of the diamonds interpreted to have originated in the transition zone and lower mantle formed from subducted carbon[9,10,11,12]. However, it is likely that a fraction of superdeep diamonds, many of which may have never reached the surface, formed during the crystallization of the mantle after the moon-forming impact in the Hadean and subsequently during the Archaean and Proterozoic eons[13]. There have been a few computational *ab initio* studies examining the behavior of carbon in silicate melts with a focus on carbon coordination by oxygen[14,15,16]; however, there have been no computational studies characterizing the genesis of diamonds in pyrolitic melts.

The analysis of diamonds in metamorphic rocks provides some information on the depths at which the diamonds formed and the mechanisms by which they grew. For example, octahedral diamonds in peridotitic and eclogitic rocks displayed a spiral growth mechanism, indicating that the crystal moved freely in carbon-bearing silicate melt[17]. The formation and growth of diamonds has been experimentally observed in a kimberlitic melt at 1800-2200 °C and 7-7.7 GPa[18], and from dolomite+iron through carbon-iron redox reactions[19]. However, the form in which carbon was dissolved in the melt and the mechanism of subsequent polymerization of carbon could not be determined due to limitations in experimental techniques.

Although redox reactions, such as the oxidation of methane or reduction of carbon dioxide, have been considered a necessary step for the formation of diamonds, it has been recently shown that diamonds can form from a reaction of carboxylates (e.g., acetate ($CH_3COO^-$)) with water at constant oxygen fugacity[8,20]. In our pyrolitic melts, we observe various oxo-carbon species, like $C_2O_2$, i.e., ethylenedione, $C_2O_4$, i.e. oxalate, (Fig. 4), suggesting that in the presence of hydrogen, the formation of diamonds from acetate is possible. These polymerized carbon chains are reminiscent of carboxylates and may be considered precursors to the formation of diamonds. This suggests a mechanism for diamond formation in pyrolitic melts at large depths that does not necessitate the direct formation of oxygen-free carbon clusters; instead diamond embryos on the nano scale could form through a pathway involving polymerized hydrocarbons.

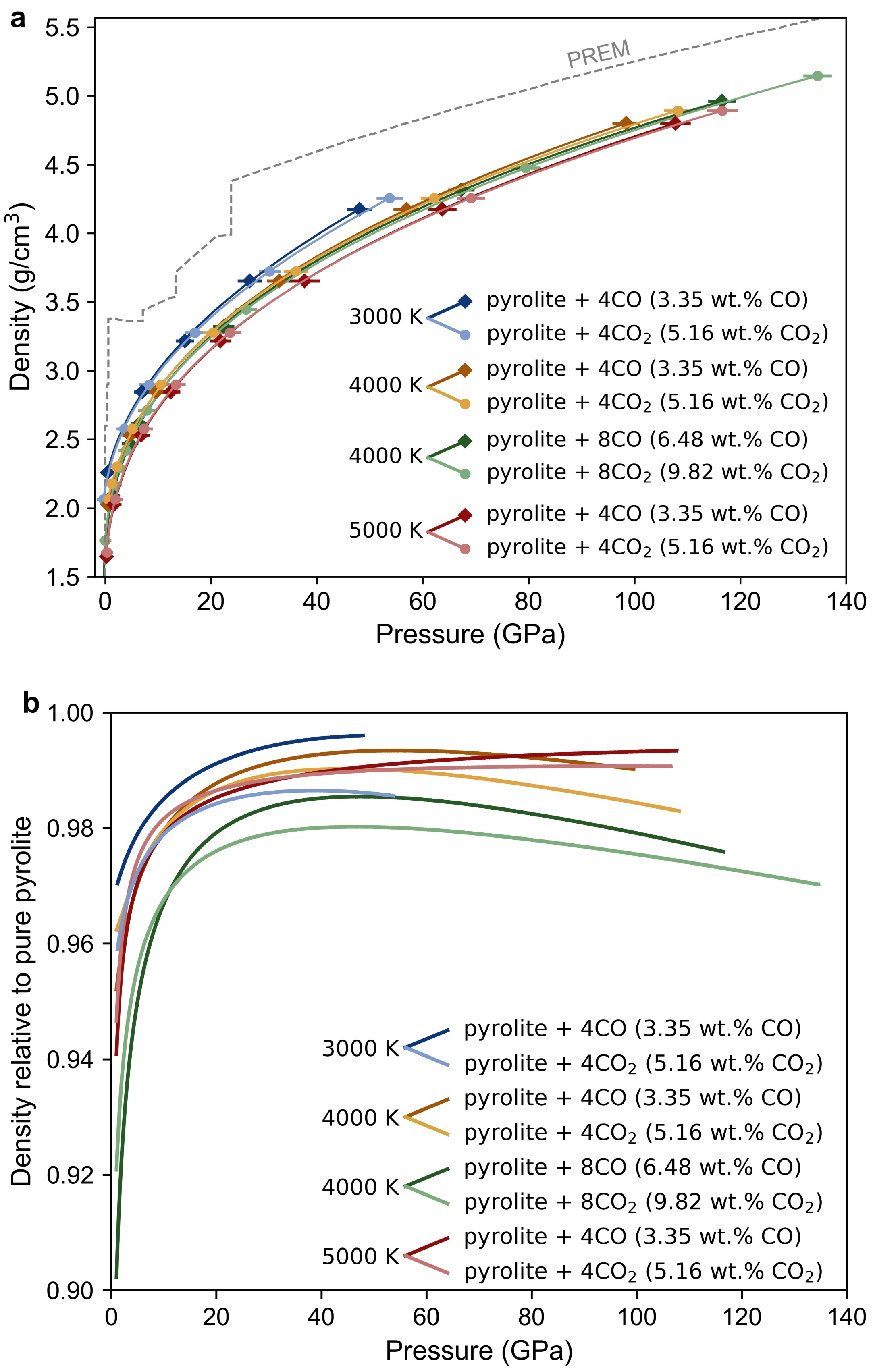
a
Density (g/cm$^3$)
Pressure (GPa)
PREM
3000 K
pyrolite + 4CO (3.35 wt.% CO)
pyrolite + 4CO$_2$ (5.16 wt.% CO$_2$)
4000 K
pyrolite + 4CO (3.35 wt.% CO)
pyrolite + 4CO$_2$ (5.16 wt.% CO$_2$)
4000 K
pyrolite + 8CO (6.48 wt.% CO)
pyrolite + 8CO$_2$ (9.82 wt.% CO$_2$)
5000 K
pyrolite + 4CO (3.35 wt.% CO)
pyrolite + 4CO$_2$ (5.16 wt.% CO$_2$)
b
Density relative to pure pyrolite
Pressure (GPa)
3000 K
pyrolite + 4CO (3.35 wt.% CO)
pyrolite + 4CO$_2$ (5.16 wt.% CO$_2$)
4000 K
pyrolite + 4CO (3.35 wt.% CO)
pyrolite + 4CO$_2$ (5.16 wt.% CO$_2$)
4000 K
pyrolite + 8CO (6.48 wt.% CO)
pyrolite + 8CO$_2$ (9.82 wt.% CO$_2$)
5000 K
pyrolite + 4CO (3.35 wt.% CO)
pyrolite + 4CO$_2$ (5.16 wt.% CO$_2$)

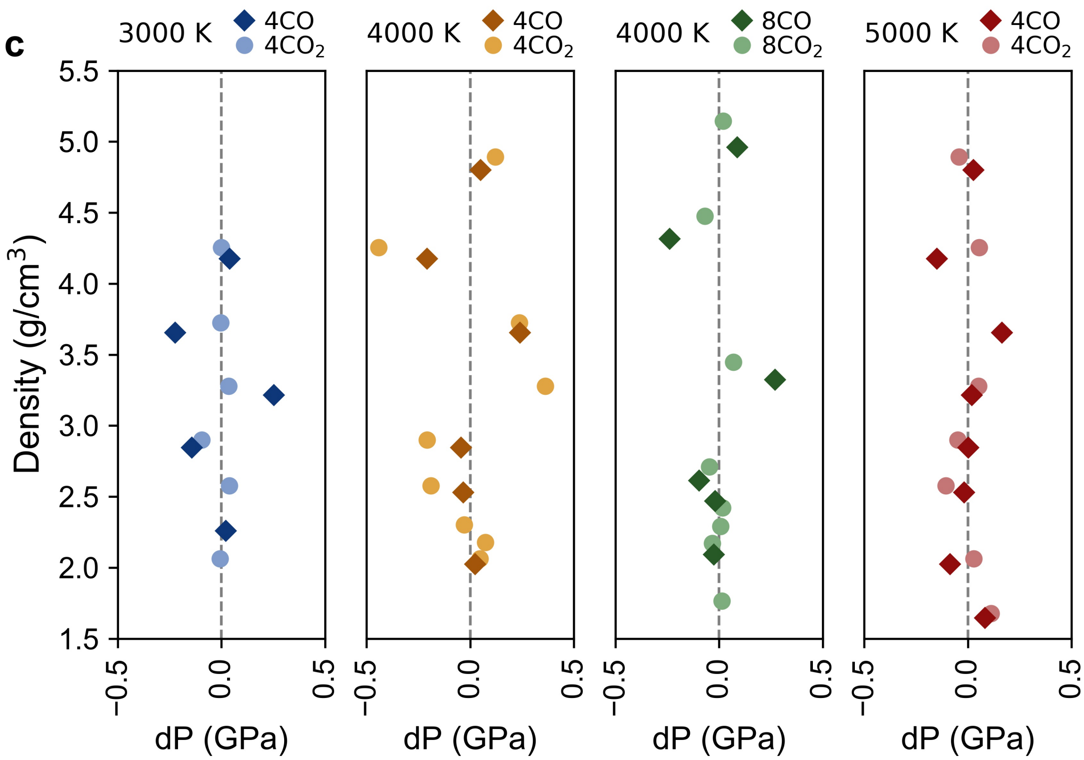


**Supplementary Figure 1 | Equation of state of carbon-bearing and carbon-free pyrolite.** (**a**) Calculated pressure-density data and the fitted fourth-order Birch Murnaghan equations of state for carbon-bearing pyrolite liquids. Pyrolite + 4CO (3.35 wt.% CO) at 3000, 4000 and 5000 K is dark red, dark orange and dark blue, respectively, while pyrolite + $4CO_2$ (5.16 wt.% $CO_2$) at 3000, 4000 and 5000 is light red, light orange and light blue, respectively. Pyrolite + 8CO (6.48 wt.% CO) and pyrolite + $8CO_2$ (9.82 wt.% $CO_2$) are dark green and light green, respectively. Error bars on pressures are standard deviations determined from the simulations. Equation of state parameters are reported in Supplementary Table 1. The Preliminary Reference Earth Model[21] (PREM) is shown as a grey dashed curve. (**b**) Density ratio of carbon-bearing pyrolite relative to pure carbon-free pyrolite at 3000 K, 4000 K and 5000 K. Unity denotes equal density. Sub-unitary values show positive buoyancy of the carbon-bearing melts with respect to carbon-free pyrolite. (**c**) Pressure residuals (dP) for the fourth-order Birch Murnaghan equation of state fits for pyrolite + 4CO (3.35 wt.% CO) at 3000 K (dark blue) and pyrolite + $4CO_2$ (5.16 wt.% $CO_2$) at 3000 K (light blue), pyrolite + 4CO (3.35 wt.% CO) at 4000 K (dark orange) and pyrolite + $4CO_2$ (5.16 wt.% $CO_2$) at 4000 K (light orange), pyrolite + 8CO (6.48 wt.% CO) at 4000 K (dark green) and pyrolite + $8CO_2$ (9.82 wt.% $CO_2$) at 4000 K (light green), and pyrolite + 4CO (3.35 wt.% CO) at 5000 K (dark red) and pyrolite + $4CO_2$ (5.16 wt.% $CO_2$) at 5000 K (light red).

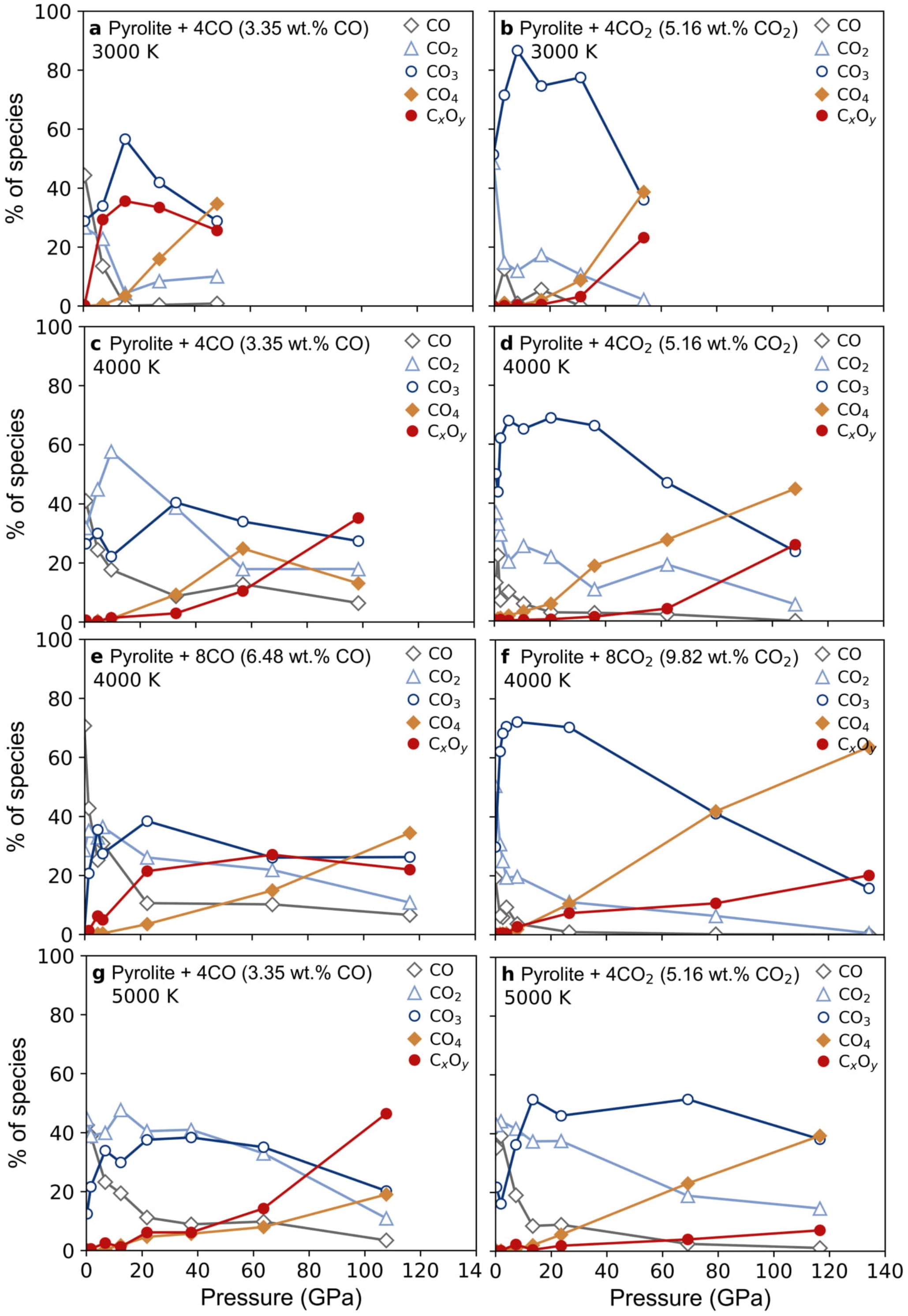


**Supplementary Figure 2 | Relative quantity of CO, $CO_2$, $CO_3$, $CO_4$ and $C_xO_y$ species as a function of pressure.** (**a**) Pyrolite + 4CO (3.35 wt.% CO) at 3000 K, (**b**) pyrolite + 4$CO_2$ (5.16 wt.% $CO_2$) at 3000 K, (**c**) pyrolite + 4CO (3.35 wt.% CO) at 4000 K, (**d**) pyrolite + 4$CO_2$ (5.16 wt.% $CO_2$) at 4000 K, (**e**) pyrolite + 8CO (6.48 wt.% CO) at 4000 K, (**f**) pyrolite + 8$CO_2$ (9.82 wt.% $CO_2$) at 4000 K, (**g**) pyrolite + 4CO (3.35 wt.% CO) at 5000 K, and (**h**) pyrolite + 4$CO_2$ (5.16 wt.% $CO_2$) at 5000 K.

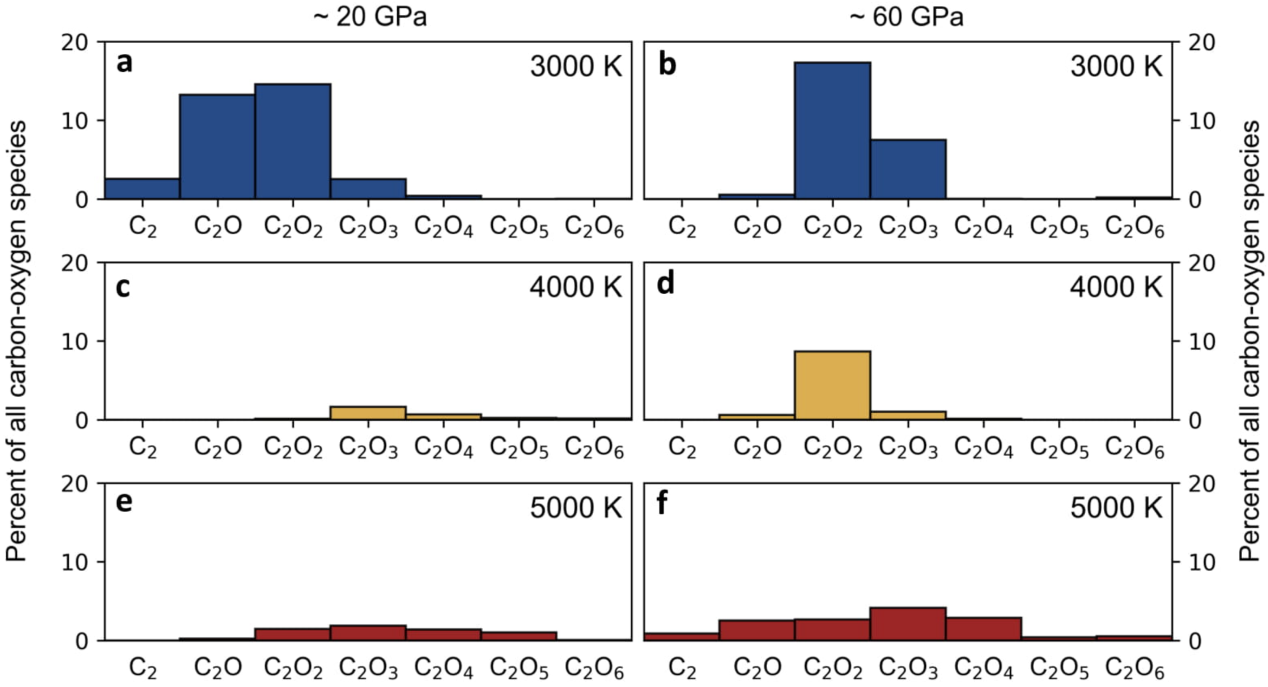


**Supplementary Figure 3 | Carbon speciation as a function of temperature.** Types of carbon species present in the pyrolitic melt + 4CO (3.35 wt.% CO) at (**a**) ~20 GPa and 3000 K, (**b**) ~60 GPa and 3000 K, (**c**) ~20 GPa and 4000 K, (**d**) ~60 GPa and 4000 K, (**e**) ~20 GPa and 5000 K, and (**f**) ~60 GPa and 5000 K. The y-axis is expressed as the percent of all carbon-oxygen species present in the melt, including unpolymerized carbon species (not shown here for clarity).

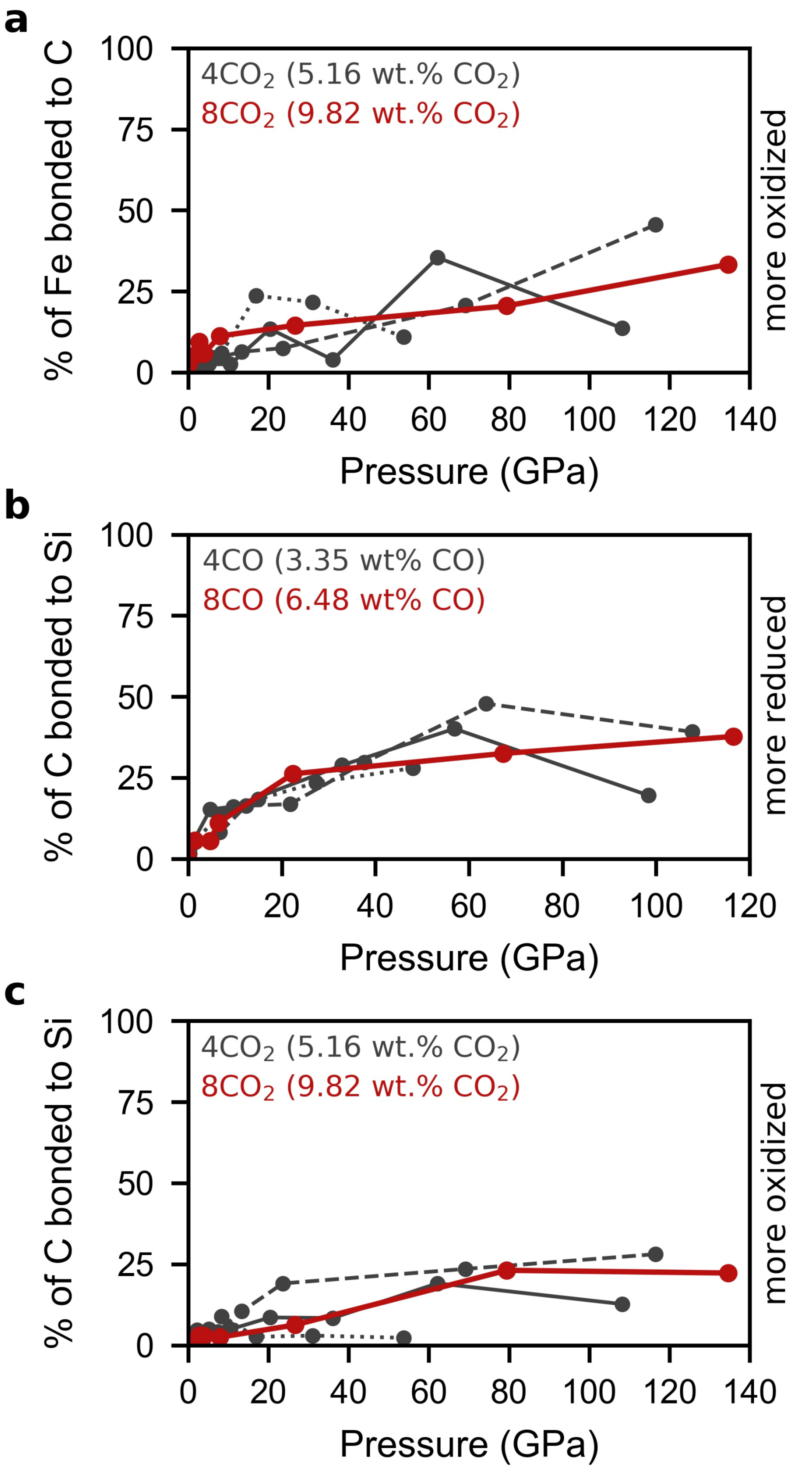


**Supplementary Figure 4 | Abundance of carbon-iron and carbon-silicon bonds.** (**a**) Amount of iron bonded to carbon as a function of pressure and temperature for the more oxidized melts (see Figure 1c in the main text for the more reduced melts). (**b**) Amount of carbon bonded to silicon as a function of pressure and temperature for the more reduced melts. (**c**) Amount of carbon bonded to silicon as a function of pressure and temperature for the more oxidized melts. For all panels, dotted lines are 3000 K, solid lines are 4000 K and dashed lines are 5000 K.

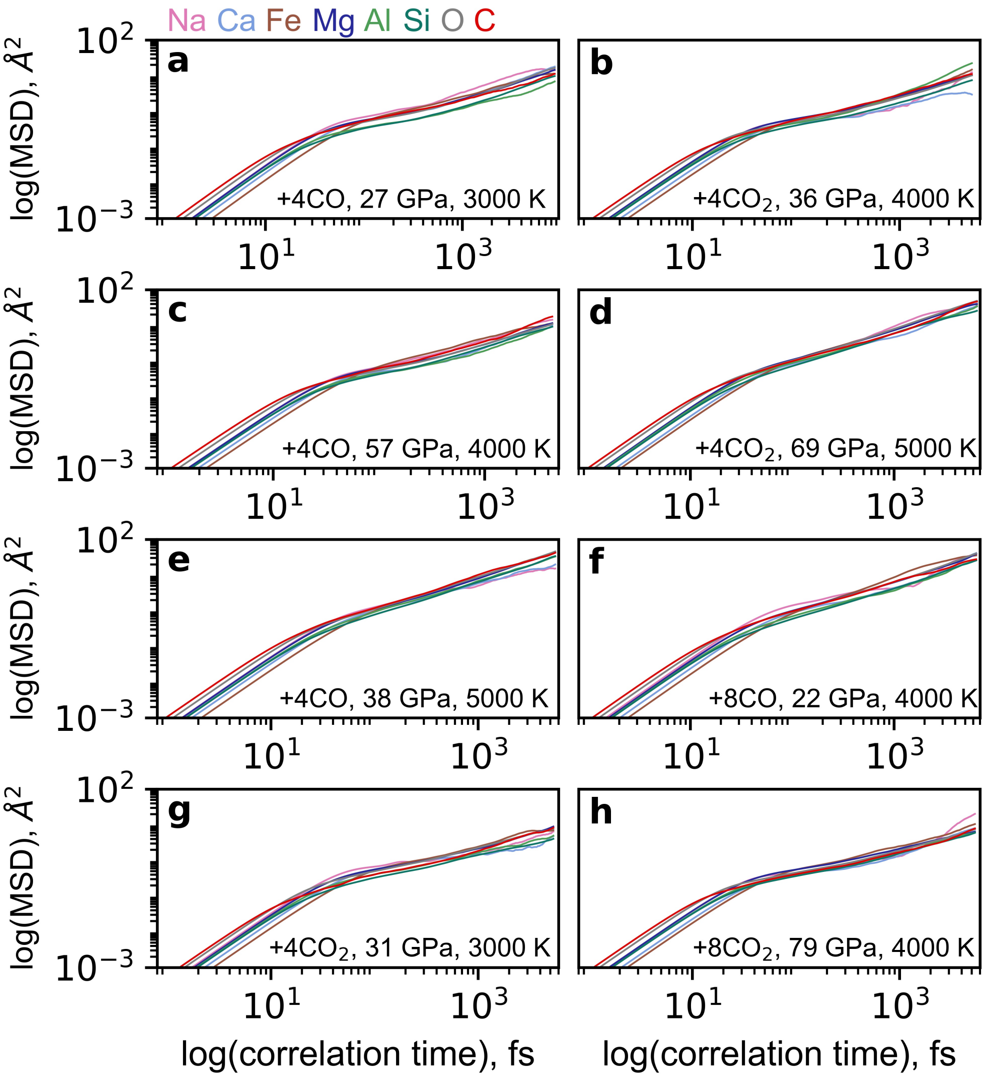


**Supplementary Figure 5 | Mean-square displacement (MSD).** (**a**) Pyrolite + 4CO (3.35 wt.% CO) at 27 GPa and 3000 K, (**b**) pyrolite + $4CO_2$ (5.16 wt.% $CO_2$) at 36 GPa and 4000 K, (**c**) pyrolite + 4CO (3.35 wt.% CO) at 57 GPa and 4000 K, (**d**) pyrolite + $4CO_2$ (5.16 wt.% $CO_2$) at 69 GPa and 5000 K, (**e**) pyrolite + 4CO (3.35 wt.% CO) at 38 GPa and 5000 K, (**f**) pyrolite + 8CO (6.48 wt.% CO) at 22 GPa and 4000 K, (**g**) pyrolite + $4CO_2$ (5.16 wt.% $CO_2$) at 31 GPa and 3000 K, and (**h**) pyrolite + $8CO_2$ (9.82 wt.% $CO_2$) at 79 GPa and 4000 K. The log of the MSD is plotted against the log of the correlation time (note: not equivalent to the simulation time) for each compositions and temperature at a range of pressures. The carbon concentration, pressure and temperature is indicated in the bottom right of each plot. The mean square displacements demonstrate that in all of the simulations, the atoms typically reach at least 10 Å$^2$. The presence of carbon in pyrolite does not affect the diffusivities of any element (see Supplementary Fig. 6). This suggests that any observed or inferred differences in viscosity between the carbon-free and carbon-bearing silicate melts should come from another mechanism, not the atomic diffusion.

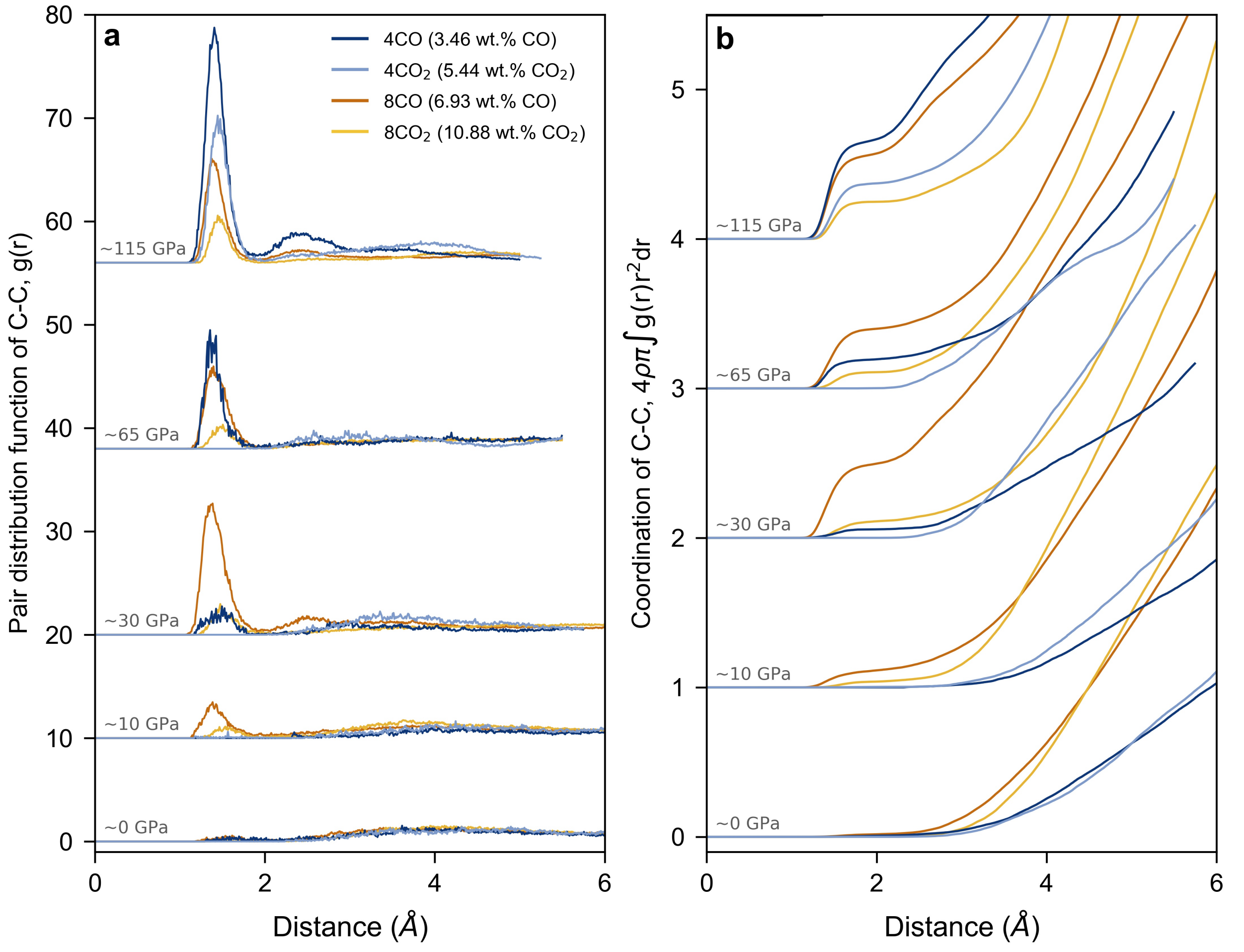


**Supplementary Figure 6 | Pair distribution functions and atomic coordinations of carbon.** (**a**) Pair distribution function for C-C atom pairs and (**b**) the coordination of C by C at 4000 K. Pyrolite + 4CO (3.35 wt. % CO) is dark blue, pyrolite + $4CO_2$ (5.16 wt.% $CO_2$) is light blue, pyrolite + 8CO (6.48 wt.% CO) is dark orange and pyrolite + $8CO_2$ (9.82 wt.% $CO_2$) is light orange. The average pressures of the four compositions are indicated to the left of each spectrum. The spectra above 0 GPa have been vertically offset for clarity.

**Supplementary Table 1 | Equation of state parameters for pyrolite with varying concentrations of carbon.** We compare our results to the *ab initio* molecular dynamics study[16] on $MgSiO_3$ melt with and without $CO_2$ and to the experimental ("exp") studies on carbon-bearing peridotitic and basaltic melts[22,23]. An isothermal fourth-order Birch Murnaghan equation of state was fitted to the pressure-density data set at each temperature for the pyrolite melts. Ref 22, ref 23 and ref 16 used an isothermal third-order Birch Murnaghan equation of state to fit their data for $MgSiO_3$, peridotite and basalt, respectively.

| Melt composition | T (K) | $\rho_0$ (g/cm$^3$) | $K_0$ (GPa) | $K'_0$ | $K''_0$ |
|---|---|---|---|---|---|
| pyrolite (C-free) | 3000<br>4000<br>5000 | 2.265(2)<br>2.03(4)<br>1.77(2) | 12.3(1)<br>8(1)<br>4.9(6) | 7.8(1)<br>8(1)<br>7.4(5) | -2.4(1)<br>-4(3)<br>-4(1) |
| pyrolite + 3.35 wt.% CO<br>(i.e., 4CO units/cell) | 3000<br>4000<br>5000 | 2.19(6)<br>1.92(4)<br>1.56(5) | 12(5)<br>7(2)<br>2.2(8) | 7(3)<br>6.7(9)<br>9(2) | -2(3)<br>-2(1)<br>-17(15) |
| pyrolite + 5.16 wt.% $CO_2$<br>(i.e., 4$CO_2$ units/cell) | 3000<br>4000<br>5000 | 2.14(1)<br>1.96(4)<br>1.56(6) | 10.0(6)<br>9(2)<br>2(1) | 7.6(4)<br>6.2(9)<br>10(3) | -2.5(5)<br>-1(1)<br>-19(24) |
| pyrolite + 6.48 wt.% CO<br>(i.e., 8CO units/cell) | 4000 | 1.71(4) | 3.3(6) | 8.2(6) | -8(3) |
| pyrolite + 9.82 wt.% $CO_2$<br>(i.e., 8$CO_2$ units/cell) | 4000 | 1.78(1) | 3.8(3) | 8.8(4) | -9(2) |
| $MgSiO_3$<br>(Ghosh et al., 2017) | 3000 | 2.56(2) | 18(1) | 6.9(2) | - |
| $MgSiO_3$ + 5.2 wt.% $CO_2$<br>(Ghosh et al., 2017) | 3000 | 2.43(3) | 16(2) | 6.5(2) | - |
| peridotite + 2.5 wt.% $CO_2$,<br>exp (Sakamaki et al., 2011) | 3000 | - | 23(1) | 7(1) | - |
| basalt + 5 wt.% $CO_2$, exp<br>(Ghosh et al., 2007) | 3000 | - | 16(1) | 5.2(2) | - |

## Supplementary References


[1] Birch, F. Finite elastic strain of cubic crystals. *Phys. Rev.* **71,** 809 (1947).
[2] Boggs, P. T. & Rogers, J. E. Orthogonal distance regression. *Cont. Math.* **112,** 183-194 (1990).
[3] Meyer, H. O. Genesis of diamond: a mantle saga. *Am. Mineral.* **70,** 344-355 (1985).
[4] Cartigny, P. et al. The origin and formation of metamorphic microdiamonds from the Kokchetav massif, Kazakhstan: a nitrogen and carbon isotopic study. *Chem. Geol.* **176,** 265-281 (2001).
[5] Kaminsky, F. Mineralogy of the lower mantle: A review of 'super-deep'mineral inclusions in diamond. *Earth Sci. Rev.* **110,** 127-147 (2012).
[6] Boyd, F. R. & Finnerty, A. A. Conditions of origin of natural diamonds of peridotite affinity. *J. Geophys. Res. Solid Earth* **85,** 6911-6918 (1980).
[7] Shee, S. R., Gurney, J. J. & Robinson, D. N., 1982. Two diamond-bearing peridotite xenoliths from the Finsch kimberlite, South Africa. *Contrib. Mineral. Petrol.* **81,** 79-87 (1982).
[8] Sverjensky, D. A. & Huang, F. Diamond formation due to a pH drop during fluid–rock interactions. *Nat. Commun.* **6,** 8702 (2015).
[9] Pal'yanov, N., Sokol, A. G., Borzdov, M. & Khokhryakov, A. F. Fluid-bearing alkaline carbonate melts as the medium for the formation of diamonds in the Earth's mantle: an experimental study. *Lithos* **60,**145-159 (2002).
[10] Walter, M. J. et al. Deep mantle cycling of oceanic crust: evidence from diamonds and their mineral inclusions. *Science* **334,** 54-57 (2011).
[11] Burnham, A. D. et al. Stable isotope evidence for crustal recycling as recorded by superdeep diamonds. *Earth Planet. Sci. Lett.* **432,** 374-380 (2015).
[12] Zedgenizov, D. A. et al. Evidence for phase transitions in mineral inclusions in superdeep diamonds of the São Luiz deposit (Brazil). *Russ. Geol. Geophys.* **56,** 296-305 (2015).
[13] Helmstaedt, H. H., Gurney, J. H. & Richardson, S. H. Ages of cratonic diamond and lithosphere evolution: constraints on Precambrian tectonics and diamond exploration. *Can. Mineral.* **48,** 6 (2010).
[14] Guillot, B. & Sator, N. Carbon dioxide in silicate melts: A molecular dynamics simulation study. *Geochim. Cosmochim. Acta* **75,** 1829-1857 (2011).
[15] Vuilleumier, R., Seitsonen, A. P., Sator, N. & Guillot, B. Carbon dioxide in silicate melts at upper mantle conditions: Insights from atomistic simulations. *Chem. Geol.* **418,** 77-88 (2015).
[16] Ghosh, D. B., Bajgain, S. K., Mookherjee, M. & Karki, B. B. Carbon-bearing silicate melt at deep mantle conditions. *Sci. Rep.* **7,** 848 (2017).
[17] Bulanova, G. P. The formation of diamond. *J. Geochem. Explor.* **53,** 1-23 (1995).
[18] Arima, M., Nakayama, K., Akaishi, M., Yamaoka, S. & Kanda, H. Crystallization of diamond from a silicate melt of kimberlite composition in high-pressure and high-temperature experiments. *Geol.* **21,** 968-970 (1993).
[19] Dorfman, S. M. et al. Carbonate stability in the reduced lower mantle. *Earth Planet. Sci. Lett.* **489**, 84-91 (2018).
[20] Sverjensky, D. A., Stagno, V. & Huang, F. Important role for organic carbon in subduction-zone fluids in the deep carbon cycle. *Nat. Geosci.* **7,** 909 (2014).
[21] Dziewonski, A. M. & Anderson, D. L. Preliminary reference Earth model. *Phys. Earth Planet. Inter.* **25,** 297-356 (1981).
[22] Ghosh, S., Ohtani, E., Litasov, K., Suzuki, A. & Sakamaki, T. Stability of carbonated magmas at the base of the Earth's upper mantle. *Geophys. Res. Lett.* **34** (2007).
[23] Sakamaki, T., Ohtani, E., Urakawa, S., Terasaki, H. & Katayama, Y. Density of carbonated peridotite magma at high pressure using an X-ray absorption method. *Am. Mineral.* **96,** 553-557 (2011).
